# *Notes from the Field: Cronobacter sakazakii* Meningitis in a Full-Term Neonate Fed Exclusively with Breast Milk — Indiana, 2018

**DOI:** 10.15585/mmwr.mm6744a7

**Published:** 2018-11-09

**Authors:** Madhura Sundararajan, Leslie A. Enane, Laurie A. Kidwell, Ryan Gentry, Stanley Danao, Samina Bhumbra, Christopher Lehmann, Megan Teachout, Jamie Yeadon-Fagbohun, Peter Krombach, Betsy Schroeder, Haley Martin, Jonathan Winkjer, Thomas Waltz, Jonathan Strysko, Jennifer R. Cope

**Affiliations:** ^1^Indiana State Department of Health; ^2^The Ryan White Center for Pediatric Infectious Disease and Global Health, Indianapolis, Indiana; ^3^Departments of Medicine and Pediatrics, Indiana University School of Medicine, Indianapolis, Indiana; ^4^Epidemic Intelligence Service, CDC; ^5^Division of Foodborne, Waterborne, and Environmental Diseases, National Center for Emerging and Zoonotic Infectious Diseases, CDC.

In January 2018, the Indiana State Department of Health (ISDH) was notified of a case of *Cronobacter sakazakii* meningitis in a female neonate who had been fed exclusively maternal breast milk. The infant was born by induced vaginal delivery at 37 weeks’ gestational age. She was discharged from the newborn nursery after 2 days and was clinically well until age 8 days, when she was admitted with poor feeding, fever of 100.4°F (38°C), and abnormal movements. Electroencephalography demonstrated multifocal seizures; MRI demonstrated multifocal restricted diffusion, leptomeningeal enhancement, and patchy hemorrhagic areas. Cultures from blood and cerebrospinal fluid yielded *C. sakazakii*, a gram-negative pathogenic bacillus. She was initially treated with meropenem, gentamicin, and antiepileptics to control seizures; when antibiotic sensitivity results were available, the antimicrobial regimen was narrowed to cefepime to complete a 21-day course. She was discharged home at age 33 days with early intervention therapies for global hypotonia and close monitoring of her development.

From birth until illness onset, the infant was fed exclusively maternal breast milk, both at the breast and expressed. Breast milk was expressed using a personal electric breast pump and was not combined with any additives such as fortifier or infant formula. The breast pump and flanges were wiped with a baby wipe after each use and occasionally cleaned with soap and water. When the bottles and pump parts were cleaned, they were disassembled, hand-scrubbed, and air-dried on a towel next to the sink; they were periodically sanitized either by boiling or in a microwave steam bag.

ISDH, in partnership with clinicians from the hospital and CDC, conducted an investigation to identify the source of infection. Items and materials tested included the cerebrospinal fluid isolate, four samples of expressed breast milk, and the breast pump kit and parts from the patient’s home. In addition, kitchen environmental surfaces were sampled using sponges that were submitted for testing. *C. sakazakii* was isolated from expressed breast milk samples, the breast pump kit, and samples obtained from the sink, drain, and drying area next to the sink. Pulsed field gel electrophoresis (PFGE) was performed on all *C. sakazakii* isolates using the PulseNet *Cronobacter* protocol (https://www.cdc.gov/pulsenet/pdf/cronbacter-pfge-protocol-508c.pdf). Environmental isolates were indistinguishable or differed by one band by PFGE from the clinical isolate.

*C. sakazakii* infection is rare and can cause sepsis and severe meningitis, associated with high morbidity and mortality, in infants fed powdered infant formula (*1*). Infection in breast-fed infants is rare but has recently been reported in two preterm neonates in association with contaminated breast pump parts (*2*,*3*). This case demonstrates the potential for invasive infection with this emerging pathogen in healthy full-term neonates fed exclusively maternal breast milk. This organism can grow rapidly in expressed breast milk without added formula (*4*). This case provides additional evidence that, although rare, expressed breast milk contaminated with *C. sakazakii* can cause life-threatening invasive infection in neonates (*2*,*3*). Although the source of contamination in this case is unknown, as was reported in the other cases, the breast pump kit became contaminated with *C. sakazakii* (*2*,*3*). Because human milk is the optimal nutrition for neonates, clinicians should proactively support and educate new parents about the importance of breast pump hygiene. Breast pump kits should be taken apart and cleaned either by hand with soap and water or in the dishwasher after every use. Parents should consider sanitizing breast pump kits daily by boiling or steaming, especially if their infant is aged <3 months, was born prematurely, or has a compromised immune system (*5*).
